# Does the Number of Oocytes Retrieved Influence Pregnancy after Fresh Embryo Transfer?

**DOI:** 10.1371/journal.pone.0056189

**Published:** 2013-02-15

**Authors:** Qianfang Cai, Fei Wan, Kai Huang, Hanwang Zhang

**Affiliations:** 1 Reproductive Medicine Center, Women and Children’s Hospital of Guangdong Province, Guangzhou, China; 2 Center for Clinical Epidemiology and Biostatistics, University of Pennsylvania, Philadelphia, United States of America; 3 Reproductive Medicine Center, Tongji Hospital, Tongji Medical College, Huazhong University of Science and Technology, Wuhan, Hubei, China; Stem Cell Research Institute, Belgium

## Abstract

**Background:**

The nature of the association between the number of oocytes retrieved and in vitro fertilization (IVF) outcomes after fresh embryo transfer remains unclear because of conflicting results reported in the studies on this subject. In addition, the influence of the quality of the embryos transferred is usually neglected. The objective of this study is to assess the relationships of the number of oocytes retrieved, the number and quality of embryos transferred, and the prospects of pregnancy after fresh embryo transfer.

**Methods:**

The data on 3131 infertile women undergoing their first IVF treatment cycle between January 2009 and December 2010 were collected retrospectively. Restricted cubic splines and stratified analyses were used to explore the relationships between the number of oocytes retrieved, the number and quality of embryos transferred, and the IVF outcomes.

**Results:**

When stratified by the number and quality of transferred embryos, no significant differences in the chances for clinical pregnancy and live birth were found in three groups of oocytes yielded (≤6, 7–14, or ≥15). The relationship between the number of oocytes retrieved and pregnancy is nearly a reflection of the pattern of the relationship between the number of oocytes retrieved and the probability of having two good-quality embryos transferred. The patients with the “optimal” number of oocytes were not only younger but also had the highest probability of having two good-quality embryos replaced.

**Conclusions:**

Similarly aged patients have similar pregnancy prospects after fresh embryo transfer when the same number and quality of embryos are replaced, irrespective of their number of oocytes. Selecting the desired number of good-quality embryos for transfer is the key to IVF success. Thus, aiming at retrieving an optimal number of oocytes to maximize IVF outcomes in a fresh cycle could place undue stress on the patients and may not be the best medical decision.

## Introduction

In 1978, the first in vitro fertilization (IVF) baby, Louise Brown, was born in the UK following a natural IVF cycle. However, normally one mature egg is produced from a natural menstrual cycle and success rates are hampered by high cancellation rates arising from unsuccessful oocyte retrieval and having no embryos available for transfer. To increase the pregnancy rates of IVF, controlled ovarian hyperstimulation (COH) was introduced in the early 1980s to stimulate multiple follicle development. This strategy enabled the retrieval of multiple oocytes at pick-up and the selection of one or more embryos for transfer. The implementation of COH has also caused significant complications, including ovarian hyperstimulation syndrome (OHSS) and high incidences of multiple pregnancies. To strike a balance between harvesting too few oocytes and reducing a couple’s chance of taking a baby home with harvesting too many oocytes and risking a negative health outcome for patients, the concept of an optimal range of oocytes retrieved has been suggested [1].

To date, the published studies on the relationship between the number of oocytes retrieved and IVF outcomes after fresh embryo transfer (ET) have presented with conflicting results [2]–[9]. The majority of studies explored the association univariately, without controlling for the number and quality of embryos transferred. As is well known, the quality of the embryos transferred is the key to IVF success. The patients with better-quality embryos transferred have higher chances of pregnancy [10]–[14]. Even in the randomized studies comparing the effectiveness of single embryo transfer (SET) versus double embryo transfer (DET), in which the treatment groups were balanced on both unobserved and observed confounding variables including maternal age and the number of oocytes retrieved, DET led to much higher success rates than SET in a fresh cycle [15]–[17]. The variables of transferred embryos are also correlated with the number of oocytes retrieved. Retrieving more oocytes enhances the selection of good embryos for transfer [11], [18]. Nonetheless, it could leave open the possibility that the effect of the number of oocytes retrieved on IVF outcomes in a fresh cycle may confound the effects of the number and quality of embryos transferred.

Different studies have shown a similar nonlinear relationship between the number of oocytes retrieved and the pregnancy rates after fresh embryo transfer. The plateau regions on the curves were used to derive the “optimal” egg number that maximizes the chance of pregnancy after fresh embryo transfer, and ovarian stimulation protocols were recommended to strive for this ideal oocytes range [6], [7]. We hypothesize that this increasing-then-plateauing shape only reflects a likelihood of transferring the maximum number of good-quality embryos, two or three in most cases, allowed under ET strategies. Neglecting information on the embryos transferred in the study population could lead to biased conclusions or misinterpretation, resulting in ill-informed decisions on ovarian stimulation and ET strategies.

Discrepancies among existing studies highlight the necessity to reinvestigate the influence of the number of oocytes retrieved on pregnancy. The present study aimed to explore the relationships of the number of oocytes retrieved, the number and quality of embryos transferred, and the probability of pregnancy after fresh ET. Specifically, the study intended to examine the hypothesis that the number and quality of embryos transferred capture the effect of the number of oocytes retrieved on pregnancy and to clarify the potential flaws of finding the “optimal” number of oocytes, according to the best pregnancy rates after fresh ET.

## Materials and Methods

### Ethics Statement

The Institutional Review Board waived the requirements for formal ethics approval of this study and patient consent because this is a retrospective analysis of conventionally treated patients, no intervention was involved except routine and standard IVF preparation and treatment, the data were anonymous, and no patient-identifying information was included.

### Patient Population

This research was a retrospective cohort study on IVF outcomes based on the medical records of patients undergoing IVF treatment at Tongji Hospital, Tongji Medical College, Huazhong University of Science and Technology, P.R. China, between January 2009 and December 2010. Only the first cycle of each patient was analyzed. Cycles involving oocyte donation, sperm donation, blastocyst transfer, in vitro maturation, unstimulated cycles, frozen embryo transfer (FET), and cycles not resulting in fresh ET were excluded from this analysis. Overall, 3131 first cycles were included in this study.

### Assessment of Embryo Quality

The embryos were classified according to three characteristics: the form of the blastomeres, the percentage of fragmentation and the cleavage rate. Good-quality embryos were defined as those that had reached the four-cell stage on day 2 or the six- to eight-cell stage on day 3, with <20% fragmentation. ET was performed on day 2 or 3 after oocyte retrieval, with a maximum of two embryos.

### Outcome Measures

The outcomes of interest were clinical pregnancy and live birth. A clinical pregnancy was confirmed through ultrasonic observation of the intrauterine gestation sac, the fetal pole and cardiac activity at 6–7 weeks of gestation. A live birth was defined as any birth event in which at least one baby was born alive and survived for more than 1 month.

### Statistical Analysis

Restricted cubic spline analysis [19] was used to explore the functional forms of relationship between the number of oocytes retrieved and IVF outcomes. To examine the hypothesis that the number and quality of embryos transferred capture the effect of the number of oocytes retrieved on IVF outcomes, we applied a two-step stratified analysis approach.

The patients with two good quality embryos transferred were divided into three subgroups by the number of oocytes retrieved: low-yield patients (≤6), intermediate-yield patients (7–14), and high- yield patients (≥15). Comparisons of the clinical pregnancy rates and live birth rates among the three groups were performed using the Pearson chi-square test. To correct for the fact that younger patients tend to have higher numbers of retrieved oocytes, the Cochran-Mantel-Haenszel (CMH) test was used to examine the association between the number of oocytes and clinical pregnancy and live birth, stratified by maternal age (<35 years, ≥35 years), among patients who had two good-quality embryos transferred.

To extend the comparisons among the three oocytes yield groups to all 3131 patients in a consistent way, we used two types of conditional logistic regression models to evaluate the association between the number of oocytes and the chance of pregnancy in a fresh cycle, controlling for effects of the embryos transferred [20]. One model was stratified by the number and quality of embryos transferred. The other model was stratified by the number and quality of embryos transferred and maternal age (<35 years, ≥35 years).

One commonly known pathway that the number of oocytes may indirectly influence pregnancy after fresh ET is that the higher the number is for the oocytes retrieved, the more likely it is that good quality embryos would be selected for ET. There may be other pathways, i.e., the number of oocytes may correlate with certain hormone level changes that may affect the endometrial receptivity of the patients. If the null hypothesis is true, the number of oocytes retrieved would not retain a significant effect in the stratified analyses after removing the effect of the embryos transferred, which would also suggest that there is no other pathway except through the embryos transferred.

The statistical analyses and plotting graphics were performed using the SAS 9.2 statistical package (SAS Inc., Cary, NC) and R statistical package (www.r-project.org).

## Results

**Figure 1:**
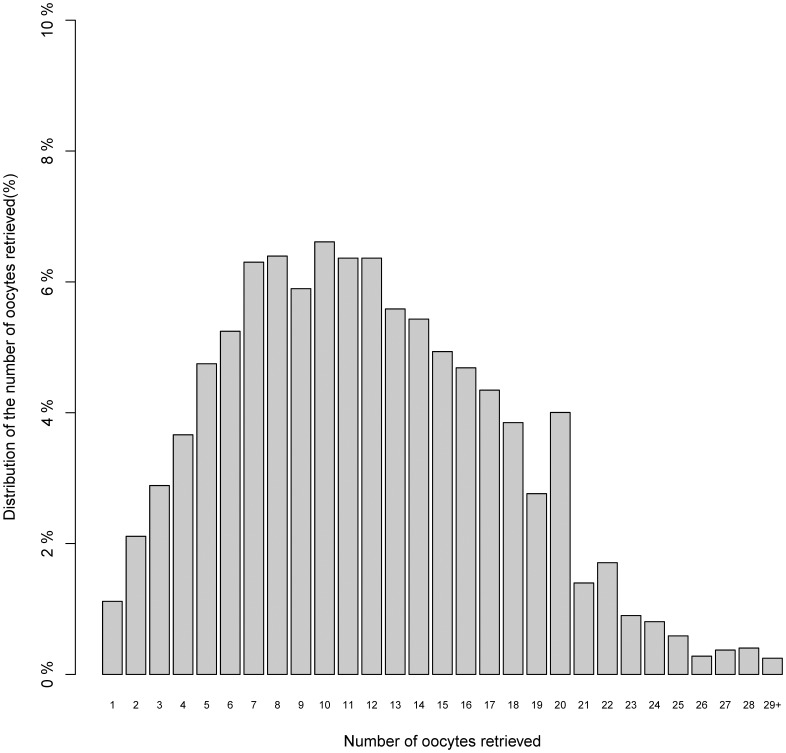
Distribution of the number of oocytes retrieved.

Table 1 shows the major characteristics of the 3131 cycles in this cohort. The distribution of the number of oocytes retrieved is presented in Fig. 1. Of the 3131 fresh non-donor IVF cycles undertaken during this study period, the rates for clinical pregnancy and live birth were 35.6% and 33.44% per ET, respectively. The clinical pregnancy rates for the three oocytes yield groups (low, intermediate, and high) were 26.8%, 37.2%, and 38.3%, respectively (P<0.0001) and for live birth rates were 26.5%, 34.3%, and 36.2%, respectively (P = 0.0002).

**Table 1:** Characteristics of the cohort (n = 3131).

Variables	n (%)
Treatment Period	
2009	1553(49.6%)
2010	1578(50.4%)
Age (year)	
mean±SD	31±3.90
≤28	924(29.51%)
(28,31]	942(30.09%)
(31,33]	493(15.75%)
(33,35]	346(11.05%)
(35,38]	286(9.13%)
>38	140(4.47%)
Duration of infertility (year)	
mean±SD	5.12±3.56
Body mass index (kg/m^2^)	
mean±SD	21.47±2.78
Insemination method	
IVF	2343(74.83%)
ICSI	608(19.42%)
50%IVF+50%ICSI	180(5.75%)
Protocol	
Long GnRH agonist protocol	2901(92.65%)
Short GnRH agonist protocol	171(5.46%)
GnRH antagonist protocol	59(1.88%)
Infertility Type	
Primary infertility	1532(48.93%)
Secondary infertility	1599(51.07%)
Diagnosis of infertility	
Tubal factor	1638(52.29%)
Male factor	463(14.8%)
Endometriosis	85(2.72%)
Ovarian factor	80(2.56%)
Unexplained	160(5.11%)
Other reasons	703(22.47%)
Number of oocytes retrieved	
median(IQR)	11.9(7–16)
≤6	589(18.81%)
7–14	1542(49.25%)
15–20	785(25.07%)
≥21	215(6.87%)
Total number of embryos	
median(IQR)	7(4–10)
≤4	973(31.1%)
5–8	1109(35.42%)
9–12	676(21.6%)
≥12	373(11.9%)

**Figure 2:**
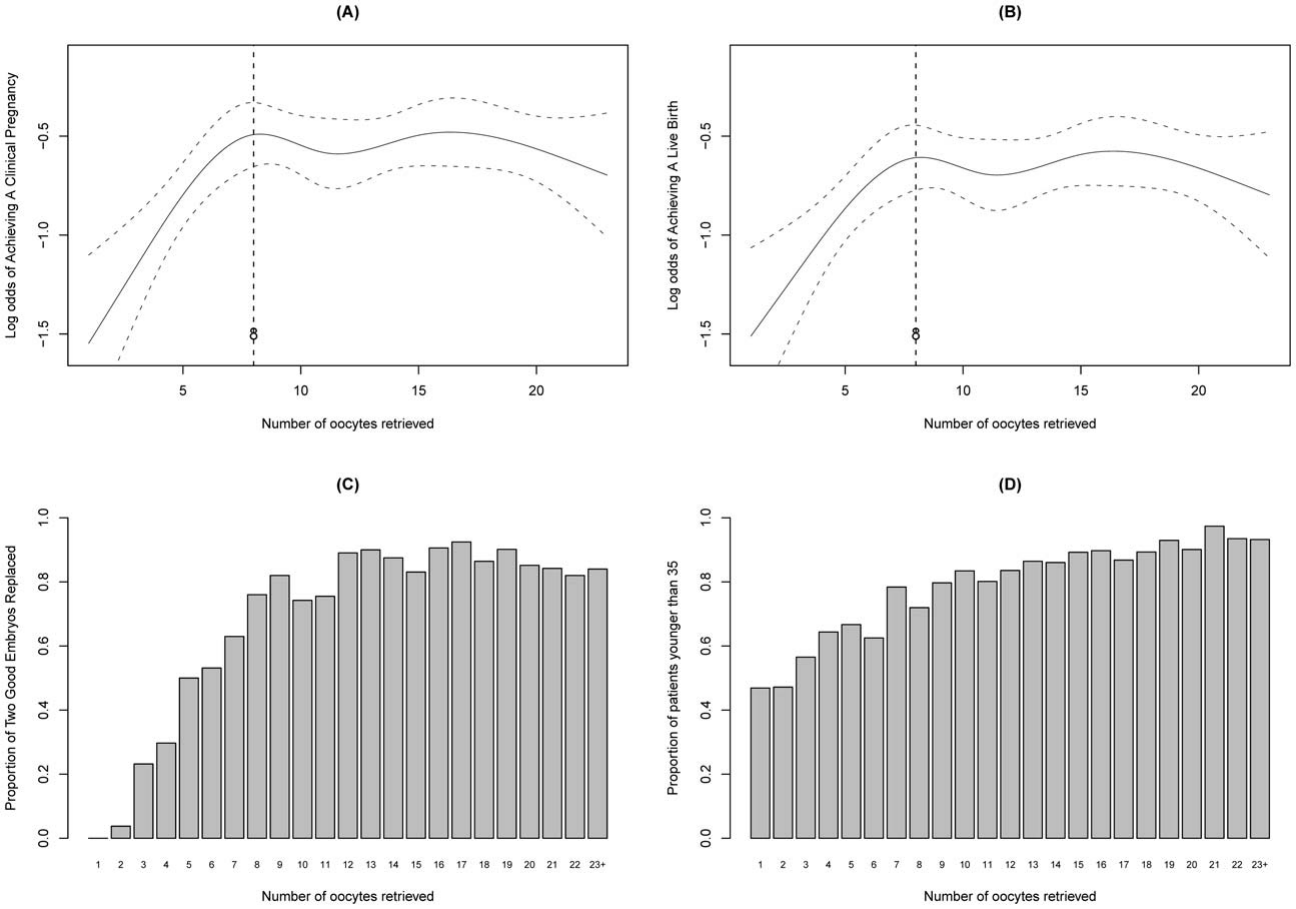
Restricted cubic spline analysis of the functional form of the association between the number of oocytes retrieved and the IVF outcomes after fresh ET. A) Restricted cubic spline curve and 95% confidence levels between the number of oocytes retrieved and the log odds of clinical pregnancy; B) Restricted cubic spline curve and 95% confidence levels between the number of oocytes retrieved and the log odds of live birth; C) Proportion of having two good-quality embryos transferred by the number of oocytes retrieved; D) Proportion of patients younger than 35 years old by the number of oocytes retrieved.

The results of the restricted cubic spline analysis are presented in Fig. 2A and Fig. 2B. The chances of clinical pregnancy and live birth rose with an increasing number of oocytes retrieved up to eight and then plateaued, which indicated that there was no association between pregnancy and the number of oocytes retrieved greater than eight oocytes because the 95% confidence bands contained a horizontal line in both scenarios. Our data did not reveal a statistically significant drop in the chances of achieving either a clinical pregnancy or a live birth among patients with more than 20 eggs (Fig. 2A and Fig. 2B). With an increasing number of oocytes, the proportion of patients having two good-quality embryos replaced during a fresh cycle increased, but this proportion was approximately equal in patients from whom more than eight oocytes were retrieved (Fig. 2C), while the proportion of patients younger than 35 years old increased steadily (Fig. 2D).

We compared the clinical pregnancy and live birth rates from cycles in which two good-quality embryos were transferred. In contrast to the results from the unstratified analysis, among these 2282 patients, clinical pregnancy rates in the low-, intermediate-, and high-yield patients (39.8%, 40.1%, and 39.1%, respectively, P = 0.907) and live birth rates (39.3%, 37.1%, and 37.0%, respectively, P = 0.826) did not differ significantly. The CMH tests for the stratified comparisons by younger and older age groups reported p-values of 0.657 and 0.512 for clinical pregnancy rate and live birth rate, respectively.

Table 2 lists the results of the stratified analysis using conditional logistic regression. The number of oocytes retrieved was not statistically significant in the conditional logistic models, but it was highly significant in the unconditional logistic regression models, without controlling for the number and quality of embryos transferred. No significant differences were found between the intermediate- (7–14) and high-yield patients (≥15) in the unconditional logistic regression, which was consistent with the graphical findings in Fig. 2A and Fig. 2B.

**Table 2:** Unconditional and conditional logistic regression analysis of the number of oocytes retrieved.

Variable		Clinical pregnancy Model						Live birth Model					
		OddsRatio^1^	P-value^1^	OddsRatio^2^	P-value^2^	OddsRatio^3^	P-value^3^	OddsRatio^1^	P-value^1^	OddsRatio^2^	P-value^2^	OddsRatio^3^	P-value^3^
Oocytes retrieved			<0.0001^a^		0.968^a^		0.693^a^		<0.0001		0.666^a^		0.396^a^
	≤6	0.59[0.47–0.73]	<0.0001	1.03[0.80–1.34]	0.809	1.10[0.85–1.44]	0.439	0.64[0.51–0.79]	<0.0001	1.09[0.85–1.42]	0.495	1.12[0.89–1.56]	0.183
	7–14	0.95[0.81–1.12]	0.562	1.02[0.86–1.20]	0.861	1.05[0.90–1.25]	0.509	0.92[0.78–1.09]	0.328	0.98[0.83–1.16]	0.804	1.02[0.86–1.21]	0.754
	≥15	1		1		1		1		1		1	

1 Unconditional logistic regression model with only the number of oocytes retrieved included.

2 Conditional logistic regression model with the number of oocytes retrieved included, stratified by the number of good-quality embryos transferred and the number of embryos transferred.

3 Conditional logistic regression model with the number of oocytes included, stratified by the number of good-quality embryos transferred and the number of embryos transferred and maternal age (<35, ≥35).

a p-value for testing overall effects of number of oocytes retrieved.

More importantly, besides no statistically significant comparisons were observed in the stratified analysis, the live birth rates and clinical pregnancy rates for the three oocytes yield groups were similar. The odds ratios reported in the conditional logistic regression models were approximately equal to one (Table 2), indicating no clinically meaningful differences in the prospects of pregnancy among the three oocytes yield groups, after controlling for the number and quality of transferred embryos and age.

## Discussion

The present study shows that the information on the number and quality of embryos transferred should not be ignored when evaluating the relationship between the number of oocytes retrieved and pregnancy in a fresh IVF cycle. There are flaws with the common practice of determining the “optimal” number of oocytes retrieved according to the maximal pregnancy rates after fresh ET. In particular, the highest pregnancy rates associated with such derived “optimal” number of oocytes retrieved relies on the underlying assumption that two good-quality embryos should be transferred among younger patients.

The way the number of oocytes retrieved affects the IVF outcomes after fresh ET is poorly understood. Some researchers have reported that the chance of pregnancy increased with an increasing number of oocytes [3], [5], [8], [9], [21]. In some of these studies, the authors used the p-values of the linear terms of the number of oocytes variable in a regression model to show the significant linear association. This method may not be accurate when the underlying functional form of the relationship is nonlinear. In contrast, some studies have graphically revealed a nonlinear association between the number of oocytes retrieved and pregnancy rates, but with different explanations [6], [7]. Moreover, the quality of the embryos transferred has been ignored in these studies, which could potentially undermine the reliability of the conclusions.

To improve on the previous studies, we not only used spline analyses to reveal the functional forms of the association between the number of oocytes retrieved and the chances of pregnancy, but we also graphically analyzed the distribution of having two good-quality embryos transferred by the number of oocytes retrieved. We found that the relationship between the number of oocytes and pregnancy in a fresh IVF cycle assumed an increasing-then-plateauing shape (Fig. 2A and Fig. 2B), which was nearly a reflection of the pattern of the association between the number of oocytes retrieved and the probability of having two good-quality embryos transferred (Fig. 2C). These findings imply that the number and quality of embryos transferred might capture the effect that the number of oocytes retrieved has on pregnancy after fresh ET. The observed non-linear relationship could primarily be attributed to the limit on the number of embryos transferred. To test this hypothesis, we performed the stratified analysis.

Our stratified analysis demonstrates that the effect of the number of oocytes retrieved on IVF outcomes is predominantly explained by the effects of the number and quality of embryos transferred. Consequently, the chances of achieving a clinical pregnancy and live birth after fresh ET are independent of the number of oocytes retrieved if the patients have the same number and quality of embryos transferred. The pattern was also revealed in a large study of 9,644 cycles by De Sutter et al. [22]. The authors observed that among patients with two good- or excellent-quality embryos transferred, poor responders had pregnancy rates similar to normal responders in both younger and older age groups. These findings also suggest that poor responders have a lower pregnancy rate than normal responders of a similar age, not because they have lower-quality oocytes but because they have fewer oocytes, which leads to fewer good-quality embryos that are transferred.

In clinical practice, ET strategies are based on a balanced consideration of success rates and the risks of multiple pregnancies and OHSS, The strategies might involve one good-quality embryo, one good-quality embryo plus a “poor”-quality embryo, two excellent-quality embryos, or other combinations. The choice of treatment directly affects the outcomes, but these decisions are not necessarily dependent on the number of oocytes retrieved from the patient.We hypothesize that two patients of a similar age have different ET strategies: for example, patient A with 15 eggs retrieved has one good-quality embryo replaced, and patient B with eight eggs retrieved has two good-quality embryos transferred. Which patient has a higher pregnancy chance? Intuitively, patient B has a better chance because it is the number and quality of embryos transferred that have a direct causal relationship with pregnancy in a fresh IVF cycle, not simply the number of oocytes retrieved.

It should be emphasized that our study does not suggest that the number of oocytes retrieved plays no role in pregnancy and live birth success. The number of oocytes could be considered a surrogate of embryo quality because the greater number of oocytes retrieved reflects a bigger pool of embryos available to select from and a higher likelihood of obtaining one or two good-quality embryos for fresh ET [11], [18]. Extra caution should be taken in attempting to use the number of oocytes to predict IVF outcomes directly and in drawing any causal conclusions. Ignoring information on the embryos transferred could lead to misinterpretation of the results.

Some investigators have reported that the relationship between the number of oocytes and pregnancy rates was hyperbolically distributed, based on the argument that ovarian stimulation and excessive response may have detrimental effects on oocyte and embryo quality, and that endometrial receptivity or supraphysiological estradiol levels could have deleterious effects on embryo implantation. These researchers then proposed the optimal number of oocytes for achieving the best pregnancy rates in IVF [6], [7]. Van der Gasst et al. found that the highest pregnancy rate was achieved with 13 oocytes, below and above which the outcomes were compromised [7]. Similarly, Sunkara et al. reported approximately 15 oocytes were required to maximize the live birth rate [6].

As shown in our study, the observed non-linear relationship is primarily attributed to the ET strategies that impose a limit on the number of embryos transferred. In general, more oocytes will increase the chances for obtaining morphologically good quality embryos. This effect has been shown to be independent of age, which subsequently improves the prospects for pregnancy [23]. In the present study, patients with more than eight eggs had a similar possibility of obtaining two good-quality embryos, the maximum number allowed for replacement. These patients’ chances of pregnancy were similar and were the highest of those analyzed (Fig. 2A, Fig. 2B and Fig. 2C).

In addition, the higher prospects for pregnancy among the patients with the “optimal” range of oocytes may partly be attributed to the effect of maternal age because younger patients tend to produce more eggs than older patients (Fig. 2D). It has been shown that the risk of chromosome abnormalities increased with advancing maternal age [24]–[26]. Even for morphologically and developmentally normal embryos selected for transfer, the prevalence of chromosome abnormalities was high in women over 35 [25]. These findings suggest that women of advanced maternal age with 15 eggs retrieved are, in general, less likely to conceive than their younger counterparts. Young poor responders might have lower chances of obtaining good-quality embryos because of the lower number of oocytes retrieved. Once good-quality embryos are available for transfer, the patients’ prospects of pregnancy after ET are not necessarily lower than those of the patients with 15 eggs. Thus, canceling cycles simply due to poor response might be a rash decision.

Another issue relating to the practice of determining the optimal number of oocytes according to the best pregnancy rates after fresh ET is that the cumulative outcome of a complete treatment cycle, rather than the outcome in a fresh cycle only, might be the more meaningful endpoint for clinicians and patients [27]. In particular, subsequent FET cycles could greatly improve the pregnancy rates of a complete treatment cycle [28].

The previous studies have suggested the use of moderate stimulation protocols over mild stimulation protocols only because fewer eggs than the derived optimal number were recovered under mild stimulation. Our study cautions against using an optimal number of oocytes retrieved as the universal standard for clinicians to plan stimulation protocols. The patients undergoing IVF are a heterogeneous group, and their responses to COH vary substantially. Other alternatives to standard COH protocols, such as mild and minimal stimulation and natural cycles, have been shown to benefit patients with poor prognosis [29]–[33]. No single approach is optimal for all patients in a population, given their considerable genetic heterogeneity. Instead of using the optimal egg number to guide the COH for maximizing pregnancy success after fresh embryo transfer, the COH approaches, as advocated by Bosch et al., should be individualized based on an assessment of each patient’s specific characteristics, including ovarian reserve, to produce an ideal number of good-quality embryos and to maximize each patient’s chance of pregnancy with minimal safety issues [2].

There are two limitations to the present study. We included few patients with more than 20 oocytes retrieved in this study (∼6.8% of all cycles). It is more likely for these patients to have one embryo replaced or to have fresh ET cancelled and all the embryos cryopreserved due to a high risk of OHSS. Therefore, we could not comment further on the slightly decreasing trend for this end point, but this limitation does not affect our major conclusions. Compared with some previously published studies, having a relatively younger study population from a single center is another limitation of our study. Further validation of the current findings with multi-center studies is necessary.

In conclusion, selecting good-quality embryos for transfer is the key to success in IVF. Although we need a balanced consideration of retrieving a reasonable number of oocytes, aiming at retrieving an optimal number of oocytes to maximize IVF outcomes in a fresh IVF cycle cannot be acted upon at present and could unnecessarily increase patients’ emotional distress.
